# Passion and perseverance: How the components of grit affect the probability of starting a business

**DOI:** 10.3389/fpsyg.2022.906701

**Published:** 2022-10-20

**Authors:** Nicolás Pablo Barrientos Oradini, Andrés Rubio, Luis Araya-Castillo, Maria Boada-Cuerva, Mauricio Vallejo-Velez

**Affiliations:** ^1^School of Administration and Business, Miguel de Cervantes University, Santiago, Chile; ^2^Faculty of Economics and Business, Andres Bello University, Santiago, Chile; ^3^Faculty of Psychology, Diego Portales University, Santiago, Chile; ^4^Human Factor, Organizations and Markets (FHOM), Faculty of Business and Economics, Universitat Rovira i Virgili (URV), Tarragona, Spain; ^5^Faculty of Psychology, University of Medellín, Medellín, Colombia

**Keywords:** entrepreneurial orientation, entrepreneurship, grit, passion, perseverance

## Abstract

There is vast evidence that accounts for the association between entrepreneurial orientation (EO) and the probability of starting a business (PSB). However, there are not many studies that test how psychological factors moderate this relationship. A variable that has been little studied in this relationship is Grit. Grit is considered a personality trait defined as perseverance and passion for long-term goals. Grit considers two sub-dimensions, one linked to the consistency of interests (Grit-Passion) and the other linked to perseverance in the effort (Grit-Perseverance). The objective of this article is to analyze the moderating role that both sub-dimensions of grit plays in the relationship between EO and PSB, considering its interaction with sociodemographic variables such as age, gender, and culture. This cross-sectional study has a sample of 1,761 participants, active workers (49.8% men; mean age 41.15 years, SD = 12.72 years; 22.9% Colombian and 77.1% Spanish). The EO scale and a Grit Scale were applied. In addition, participants were asked, based on their perception, how likely they were to start their own business within the next 5 years. A simple moderation analysis was considered to test the moderating role of grit in the relationship between EO and PSB. Subsequently, a double moderation analysis was carried out in order to identify which sociodemographic variables moderate the moderating effect of grit on the relationship in question. The results show that only the Grit-Passion component of grit moderates the relationship between EO and PSB. Regarding the sociodemographic variables, neither age, culture, nor gender showed a moderating effect on the moderation exercised by Grit-Passion in the relationship between OE and PSB. The results are discussed in terms of psychological capital, particularly with an emphasis on explaining why only the Grit-Passion shows a moderating effect on the relationship between EO and PSB, in detriment of Grit-Perseverance. In addition, the power of grit in the field of entrepreneurship is discussed, considering that its moderating effect is transversal to variations in age, gender and culture, as well as its relevance when considering interventions and pedagogical models in the field of entrepreneurship.

## Introduction

Entrepreneurship, understood as the human activity oriented to the design, development, and execution of projects that create value (economic or social), from problems and/or opportunities identified in the market (or in society, in general), is constituted as a relevant economic and social phenomenon, as it allows individuals to develop in economic and psychological aspects (creativity, achievement) and, at the same time, contribute to society in economic development and value generation (Quadrini, 2000; Lippmann et al., 2005; Avila, 2021). Some authors suggest that entrepreneurship corresponds to one of the main vehicles of social mobility (Pathak and Muralidharan, 2018), while others highlight its value in terms of personal fulfillment and life satisfaction (Brieger et al., 2020). Considering the above, studying the variables associated with entrepreneurship is of great interest, since it allows us to better understand this phenomenon and also promote it. Within the variables related to entrepreneurship, the one that appears to be most directly related to entrepreneurship or the ability to undertake is entrepreneurial orientation (EO).

In this sense the association between EO and specific actions to set up a business or the Probability of Setting up a Business (PSB) has been extensively studied (Rauch et al., 2009; Vij and Singh Bedi, 2012; Cho and Lee, 2018). However, some psychological factors that could affect this relationship and that could help better understand its nature, have not yet been sufficiently explored within the entrepreneurial field.

One of these factors is grit (Al Issa, 2020). Grit is defined as trait-level passion for and perseverance in pursuing long-term goals. It is considered one of the most relevant personality traits when explaining the variety of responses to adversity or failure experienced by people who are involved in projects in the longer term (Duckworth et al., 2007). While some people tend to persevere in the pursuit of their goals (despite the barriers, obstacles and failures that appear along the way), other people tend to abandon them when they become challenging. This is precisely what the construct of grit apprehends.

Theoretically, the construct of grit has been defined from two sub-dimensions. On the one hand, there is passion, which refers (in the context of grit) to the tendency to remain committed to the same goals for months and years or, in other words, to have consistency in long-term interests. On the other hand, there is perseverance of effort, which refers to the tendency to work diligently toward the objectives, despite the obstacles that arise (Duckworth et al., 2021).

Different studies show that grit is a personality trait associated with positive emotional-motivational states that are associated, at the same time, with the achievement of long-term goals. In particular, grit is positively associated with self-efficacy (Alhadabi and Karpinski, 2020), resilience (Karaman et al., 2019), growth mindset (Lee and Jang, 2018), self-determination (Kim et al., 2021), motivation (Jordan et al., 2019), self-control (Duckworth and Gross, 2014), and leadership (Samborowski et al., 2021), among others constructs. These associations could give rise to think that grit could be positively associated with EO, entrepreneurial behaviors and affect the relationship between both variables.

We take into account that the possible effects that grit could have on the relationship between OE and more specific entrepreneurial behaviors (such as PSB) have not been analyzed. Furthermore, we can see that grit stands out as a construct of interest when it comes to understanding phenomena where variables such as motivation, effort, focus, and perseverance are key (as is the case of entrepreneurship). Considering the above, the objective of this study is to address this knowledge gap by analyzing the moderating effect of the grit subcomponents (passion and perseverance) on the relationship between EO and PSB. Specifically, to analyze how the different levels of passion and perseverance shown by individuals can affect the relationship between their EO and PSB. Likewise, this study also seeks to analyze whether the possible moderators found are moderated by sociodemographic factors. This in order to identify whether the effect of grit on the relationship between EO and PSB is general or depends on variables such as age, gender, or culture.

The relevance of addressing this knowledge gap lies in the identification of key variables that allow increasing the probability of moving from attitudes related to entrepreneurship to specific entrepreneurial behaviors. In this way, these variables could be considered when identifying profiles of effective entrepreneurs, as well as to develop pedagogical content in the training of new entrepreneurs. In the case of grit, it is important to know if, in particular terms, passion and/or perseverance play a key role in this context, in order to identify increasingly specific characteristics of individuals that are favorable to entrepreneurship, to focus on them and promote them.

The present study aims to analyze the relationship between the variables of EO, PSB, and the types of grit. First, the methodological aspects that frame the study will be described. Subsequently, the relationship between EO and PSB will be analyzed, as well as the relationship of these two variables with the types of grit. Once this is done, the possible moderating effect of grit types on the relationship between EO and PSB will be analyzed (also analyzing whether sociodemographic variables affect this moderation). Finally, the results will be discussed, and the conclusions and limitations of the study will be presented, as well as future lines of research.

## Materials and methods

### Participants

This quantitative study with a cross-sectional design is based on a convenience sample of 1,761 participants of active workers contacted during 2018 and 2019. The average age of the sample was 38.88 years (SD = 12.53 years), with 49.8% of the sample being male (50.2% female), 22.9% Colombian nationals and 77.1% Spanish nationals.

### Measures

The instrument considered, among other scales, an EO Scale and a Grit Scale, and also an item evaluating their perception of the probability of starting a business in the next 5 years.

#### Entrepreneurial orientation scale

Adapted to Spanish by Boada-Grau et al. (2016) based on its original version, developed by Lee et al. (2011), it is a Likert-type scale with 12 items. The scale measures the degree of agreement or disagreement on different levels (on a scale of one to five, with 1 = completely disagree, and 5 = completely agree). These assess EO in four sub-dimensions: Autonomy (related to self-sufficiency in facing challenges), innovation (related to enjoyment and orientation to work on new things), risk-taking (related to the willingness to face difficulties in the future), and aggressive competitiveness (related to perseverance and belief in success). Examples of sentences considered in the items are “I don’t want to be financially supported by my parents, family, etc., because I am already an adult,” “I am more interested in starting my own business than in getting a job,” and “even if I start new businesses and fail many times, I will keep trying until I succeed.” According to Boada-Grau et al. (2016) the instrument had good psychometric properties, both in terms of validity and reliability. In this study, the overall reliability of the instrument, as shown by its Cronbach’s Alpha, was 0.74. The sum of the scores obtained in each of the 12 items that make up the scale was used to calculate the total score of the scale.

#### Grit scale

The original version of the grit scale was developed by Duckworth et al. (2007), which was adapted to Spanish and validated by Barriopedro et al. (2018). This scale has 12 items that measure perseverance and passion for long-term goals from two sub-dimensions (each one made up of six items): consistency of interests (passion) and perseverance of effort. The passion subdimension includes items such as “New ideas and new projects sometimes distract me from previous ones” and “I often set a goal but later choose to pursue a different one” which must be evaluated on a scale from 1 to 5, according to their degree of agreement/disagreement with the statements (1 = completely disagree, 2 = somewhat disagree, 3 = neither agree nor disagree, 4 = quite agree, and 5 = completely agree). The perseverance sub-dimension includes items such as “I finish whatever I begin” and “I have achieved a goal that took years of work,” which should be evaluated in the same way. The authors of the Spanish adaptation reported good psychometric properties, considering both its validity and reliability. In this study, reliability (Cronbach’s alpha) was 0.70 for the passion sub-dimension and 0.71 for the perseverance sub-dimension. The total score for each dimension of the scale was calculated based on the sum of the scores obtained in each of the five items that make up the scale. In the case of the items of the passion sub-dimension, the score of the items was inverted, because they referred to exactly the opposite that the construct theoretically referred to.

#### Probability of starting a business and sociodemographic factors

The participants’ perceived likelihood of starting their own business in the next 5 years was measured using the statement “estimate the probability of starting your own business in the next five years” (PSB), which could be rated on a scale from 0 to 10, where 0 corresponded to “not at all,” 5 to “neutral,” and 10 to “very likely.” In terms of sociodemographic factors, participants were asked their age (in years) and gender (male/female). To assess the culture of the participants (Latin American or European), the country where the instrument was applied (Colombia/Spain) was taken into account.

### Data analysis

First, descriptive analyses were carried out for all the variables involved in the study (calculation of minimum, maximum, mean, and standard deviations). Also, a bivariate correlation analysis (Spearman’s rho) was then performed among all the variables, in order to observe how they were associated. Next, two simple moderation analyses were carried out to test the moderating role of both sub-dimensions of grit in the relationship between EO and PSB. Then, a double moderation analysis was carried out in order to determine which sociodemographic variables (age, gender, and culture) moderates the moderating effect of grit in the relationship between EO and the perceived possibility of setting up a business. The statistical analyses were carried out using the IBM-SPSS v.24 program and the PROCESS for SPSS v3.2.01 modelling tool (Hayes, 2018).

## Results

### Descriptive results

Table 1 shows the minimum and maximum scores, the mean, and the standard deviation of each of the variables considered in the study. To facilitate reporting of results, the passion sub-dimension of grit will be named Grit-Passion and the perseverance of effort sub-dimension of grit will be named Grit-Perseverance.

**TABLE 1:** Descriptive analysis of the study variables.

	Min.	Max.	*M*	*SD*
1. EO	12.00	60.00	38.68	7.69
2. Grit-Passion	6.00	30.00	17.60	4.30
3. Grit-Perseverance	6.00	30.00	22.63	3.92
4. PSB	0.00	10.00	4.72	2.56

Table 2 presents the Spearman correlation coefficients (Spearman rho), among all the variables. As it can be observed, all associations were positive and statistically significant and the Grit-Perseverance showed a stronger association than the Grit-Passion, with both EO and PSB.

**TABLE 2:** Bivariate correlation matrix (Spearman’s rho) for the study variables.

	1	2	3	4
1. EO	1			
2. Grit-Passion	0.27**	1		
3. Grit-Perseverance	0.35**	0.09*	1	
4. PSB	0.53**	0.12**	0.31**	1

**The correlation is significant at the 0.01 level (bilateral). *The correlation is significant at the 0.05 level (bilateral).

### Simple moderation analysis

This section presents the results of the simple moderation analyses performed. These models considered PSB as a dependent variable, EO as an independent variable and the Grit-Passion and Grit-Perseverance as moderating variables. A BCa bootstrapped CI based on 5,000 samples was used to calculate the confidence intervals of all the models used. The mean, low, and high values of the moderating variables considered their mean plus/minus a standard deviation.

#### Grit-Perseverance as a moderator of the relationship between entrepreneurial orientation and perceived probability of starting a business

Table 3 shows the results of the linear regression model that considers PSB as a dependent variable, and EO, Grit-Perseverance, and the interaction between them, as independent variables.

**TABLE 3:** Linear model of predictors of PSB, considering Grit-Perseverance (*R*^2^ = 28.08%, *p* < 0.001).

	*B*	95% CI	SE B	*t*-value	*P*-value
Constant	−0.42	[−3.15, 2.31]	1.39	−0.30	0.76
EO	0.12	[0.05, 0.19]	0.04	3.31	<0.01
Grit-Perseverance	0.06	[−0.06, 0.19]	0.06	0.99	0.32
EO × Grit-Perseverance	0.00	[0.00, 0.00]	0.00	0.55	0.58

In this case, the interaction was not statistically significant (*p*-value = 0.58). That is, there is no evidence to argue that the Grit-Perseverance moderates the relationship between EO and PSB.

#### Grit-Passion as a moderator of the relationship between entrepreneurial orientation and perceived probability of starting a business

Table 4 shows the results of the linear regression model that considers PSB as a dependent variable, and EO, Grit-Passion, and the interaction between them, as independent variables.

**TABLE 4:** Linear model of predictors of PSB, considering Grit-Passion (*R*^2^ = 26.11%, *p* < 0.001).

	*B*	95% CI	SE B	*t*-value	*P*-value
Constant	−2.13	[−4.54, 0.27]	1.23	−1.74	0.08
EO	0.21	[0.15, 0.27]	0.04	7.09	<0.001
Grit-Passion	0.14	[0.00, 0.28]	0.07	2.00	<0.05
EO × Grit-Passion	0.01	[0.00, 0.01]	0.00	1.98	<0.05

**FIGURE 1:**
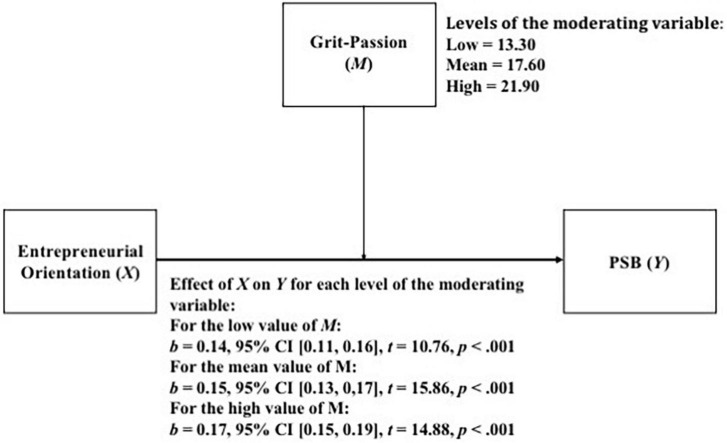
Simple moderation model considering Grit-Passion as moderator.

The fact that the interaction between independent variables was statistically significant for the model (*p*-value < 0.05) means that the moderation is statistically significant. An analysis was then carried out how the relationship between EO and PSB varied for the different levels of Grit-Passion. The results of this analysis are presented in Figure 1. As shown, as the Grit-Passion increases, the relationship between EO and PSB becomes stronger.

### Double moderation analysis

**FIGURE 2:**
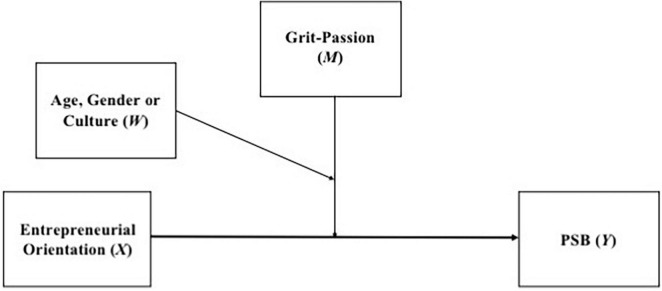
Conceptual model of double moderation analysis.

The results of the double moderation analyses are presented below. They were performed to observe how age, gender, and culture, can moderate the moderation of Grit-Passion in the relationship between EO and PSB (see Figure 2).

**FIGURE 3:**
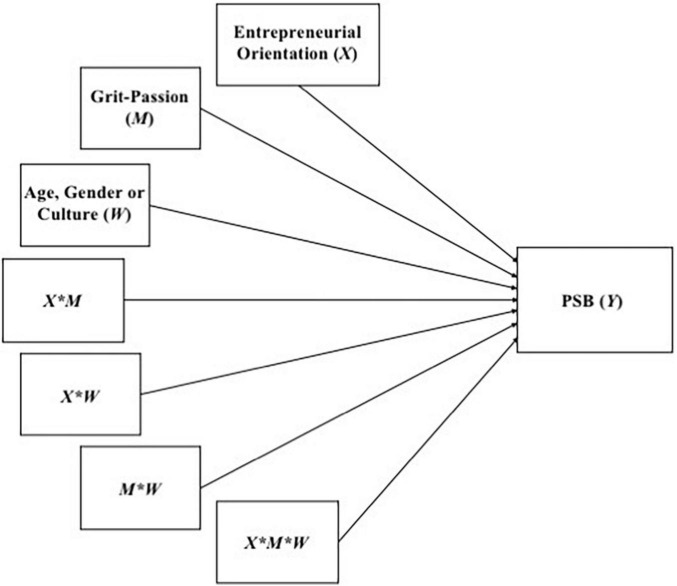
Multiple linear regression analysis for the double moderation model. *Interaction between variables.

This procedure considers a multiple linear regression analysis, which includes as a dependent variable the PSB, and as independent variables the EO, Grit-Passion, one of the sociodemographic factors (age, gender, or culture), and all possible combinations between these three variables (including the triple combination), as presented in Figure 3.

As in the previous analyses, a BCa bootstrapped CI based on 5,000 samples was used to calculate the confidence intervals of all the models used. Due to the quantity and complexity of the models, only the results of the triple interaction between the EO, the Grit-Passion, and each of the sociodemographic factors are presented, as this is the only factor that can show if the double moderation is statistically significant. These results are presented in Table 5.

**TABLE 5:** Statistical significance of the interaction between EO, Grit-Passion, and sociodemographic factors.

	*B*	95% CI	SE B	*t*-value	*P*-value
EO × Grit-Passion × Age	0.00	[0.00, 0.00]	0.00	−1.33	0.18
EO × Grit-Passion × Gender	0.00	[−0.01, 0.01]	0.00	0.28	0.78
EO × Grit-Passion × Culture	0.00	[0.00, 0.01]	0.00	0.92	0.36

Regarding the sociodemographic variables, neither age, culture, nor gender showed a moderating effect on the moderation exercised by Grit-Passion in the relationship between EO and PSB. In other words, the strength with which Grit-Passion moderates the relationship between EO and PSB does not vary according to the number of years the individuals are, their gender, or their culture.

## Discussion

The objective of this study was to address the way in which a psychological variable can mediate the relationship between EO and PSB. The results of this article complemented those presented in the paper “Curiosity as a Moderator of the Relationship between EO and Perceived Probability of Starting a Business” (Barrientos-Oradini et al., 2022). Both articles are part of the same investigation, which aims to contribute to the identification of differentiating psychological factors in the process of moving from an EO to more specific entrepreneurial behaviors and contribute to the “Entrepreneurial Psychological Capital and Spirituality: a core distinction among entrepreneurs” research topic. The same sample and database were used, but at the time of analysis both studies were carried out independently.

Considering that there was not much information regarding the moderating role of Grit in EO and actual PSB, the aim of this study was to analyze this possible moderating role for the two sub-dimensions of the Grit construct. One of them is passion, which refers (in the context of grit) to the tendency to remain committed to the same goals for months and years or, in other words, to have consistency in long-term interests. And the other is perseverance of effort, which refers to the tendency to work diligently toward the objectives, despite the obstacles that arise (Duckworth et al., 2021).

This study showed that only the Grit-Passion moderated the relation between the EO and the PSB. The higher the levels of Grit-Passion, the stronger the relation between the EO and PSB. This moderating effect could be explained by the consistency of interests allowing individuals to remain focused (or with controlled dispersion), which would have positive effects on task achievement. Added to this is the fact that long-term focus is positively associated with entrepreneurial behavior (Thapa and Shah, 2021). In this sense, having higher levels of Grit-Passion would allow individuals to carry out tasks more effectively.

Moreover, the fact that Grit-Perseverance did not moderate the relationship could be explained because perseverance of effort alone is not a guarantee of achieving more specific entrepreneurial behaviors. It could be that the effort, haphazardly, very dispersed, and attacking different things at the same time, could fulfill the saying “he who takes ahold of too much can’t hold on to all of it.” It is, precisely, the ability to maintain focus on a challenge (Grit-Passion) what makes the difference when developing an enterprise effectively.

In addition, the aim was also to study how sociodemographic factors, such as age, gender, and culture, might moderate the moderation in question. This was only analyzed for Grit-Passion, as it was the only one that showed a statistically significant moderation of the relationship between EO and PSB. The results showed that neither age, nor gender, nor culture (Spain or Colombia) showed a moderating effect on the moderation exerted by Passion-Gritt on the relationship between EO and PSB. This means that the influence of Grit-Passion on the relationship between EO and PSB is transversal to whether the individuals are young or old, men or women, and Spanish or Colombian. This is interesting, since it could show the strength that Grit-Passion has when it comes to influencing the exercise of specific entrepreneurial behaviors, regardless of the sociodemographic characteristics of the individuals in question.

## Conclusion

The results of this study can be framed within the field of entrepreneurial psychological capital. Psychological capital is defined as a positive psychological state that is mainly based on four elements: self-efficacy, optimism, hope, and resilience (Newman et al., 2014). If psychological capital is applied to the field of entrepreneurship, it is possible to see how certain psychological characteristics can be part of the psychological resources people have in order to successfully face the challenges of entrepreneurship. Some studies show that psychological capital is positively associated with entrepreneurs’ business success (Baluku et al., 2016; Wang et al., 2018). Baron et al. (2016) also demonstrated that high levels of psychological capital lead to low levels of stress in entrepreneurs, while Wang et al. (2018) showed how psychological capital is associated to entrepreneurs’ job satisfaction. In turn, Grit-Passion can be associated with elements of psychological capital such as self-efficacy, considering the determination implied by Grit-Passion to achieve what one sets out to achieve with respect to specific objectives; optimism and hope, considering that the idea of staying focused could go hand in hand with the feeling of a good forecast; and resilience, considering that maintaining a constant interest in a specific objective goes hand in hand with the ability to overcome the adverse circumstances that it presents.

As pointed out in the introduction, the results of this article are relevant in the sense that they can help us identify profiles of successful individuals in the field of entrepreneurship. Likewise, identifying Grit-Passion as a relevant variable when promoting more concrete actions in the field of entrepreneurship, serves as an input to design pedagogical interventions that aim to develop or strengthen the idea of the consistency of interests in children, adolescents, and adults (Christopoulou et al., 2018).

## Data availability statement

The raw data supporting the conclusions of this article will be made available by the authors, without undue reservation.

## Ethics statement

Ethical review and approval was not required for the study on human participants in accordance with the local legislation and institutional requirements. The patients/participants provided their written informed consent to participate in this study.

## Author contributions

NB and AR: preparation of the theoretical framework, methodological framework, and data analysis. LA-C, MB-C, and MV-V: data collection, database construction, discussion elaboration, and general revision of the manuscript. All authors contributed to the article and approved the submitted version.
